# Chronic Disease in Low-Resource Settings: Prevention and Management Throughout the Continuum of Care—A Call for Papers

**DOI:** 10.3390/ijerph20043580

**Published:** 2023-02-17

**Authors:** Martin Heine, Susan Hanekom

**Affiliations:** 1 Julius Global Health, Julius Center for Health Sciences and Primary Care, University Medical Center Utrecht, Utrecht University, 3584 CX Utrecht, The Netherlands; 2 Institute of Sport and Exercise Medicine, Department of Exercise, Sport and Lifestyle Medicine, Faculty of Medicine and Health Sciences, Stellenbosch University, Stellenbosch 7602, South Africa; 3 Division of Physiotherapy, Department of Health and Rehabilitation Sciences, Faculty of Medicine and Health Sciences, Stellenbosch University, Stellenbosch 7602, South Africa

## 1. Burden of Disease and Multimorbidity

Multimorbidity, defined as the presence of two or more chronic conditions in an individual, has become a global public health challenge [1]. The increasing burden of chronic diseases has resulted in a growing number of individuals living with multiple conditions, leading to complex and intertwined health issues. Multimorbidity has significant impacts on the individual’s quality of life, healthcare utilization, and treatment outcomes. The prevalence of multimorbidity is particularly high in low-income and middle-income countries, where the burden of both communicable and non-communicable diseases is substantial [2,3]. Estimates for the prevalence of multimorbidity in low-to-middle-income countries range from 13 to 87% [2,3], while adverse social determinants of health further exacerbate that risk [4,5]. It is well described that nearly 80% of premature mortality due to chronic disease affects low-to-middle-income countries [6]. In these environments, the limited resources available for healthcare and social support systems exacerbate the challenges faced by individuals with multimorbidity [7]. These populations are also more vulnerable to the development of multiple chronic conditions due to a higher prevalence of risk factors such as poverty, poor nutrition, and limited access to health education and services [4,5]. Chronic diseases often include mental health deficits and occur in cluster patterns rather than randomly [8]. Notably, different clusters of chronic disease or diseases with chronic sequela tend to intersect in low-resource settings, when compared to high-resource environments. The presence of multimorbidity increases the risk for impairments in function, limitations in activity, restrictions in participation, and reduction in health-related quality of life (including mental health) [9]. Hence, a strong(er) focus on multimorbidity is overdue and essential to successfully improve global health outcomes; however, it requires additional consideration in terms of healthcare innovation.

## 2. Continuum of Care

Continuum of care is a concept involving an integrated system of care that guides and tracks patients over time through a comprehensive array of health services spanning all levels of intensity of care [10], with functional linkages between levels of care in the health system and between service–delivery packages so that the care provided at each time and place contributes to the effectiveness of all the linked packages [11]. 

The behavioral health continuum of care model, first introduced by the Institute of Medicine (1994), recognizes four main “opportunities” in addressing (behavioral) health.

- Health promotion, as strategies designed to create environments and conditions that support health and the ability of an individual to withstand challenges. Such strategies can also reinforce health services further down the care continuum.
- Prevention, as universal, selective, or indicated strategies or interventions prior to the onset of disease. These interventions are intended to prevent or reduce the risk of developing a health problem.
- Treatment, as services provided to an individual with a health problem. This includes strategies for efficient case identification (e.g., hypertension screening) as well as standard (ideally evidence-based) treatments for known health conditions or risk factors.
- After-care, as services to support individuals’ abilities to live productive and fulfilling lives despite the (chronic) health problem or sequelae. These interventions include strategies to promote compliance with long-term treatment with the aim to reduce the risk for recurrence or multimorbidity), as well as rehabilitation services aimed at mitigating disease impact in body function (impairment), activity (limitations), participation (restrictions), and health-related quality of life.

An important feature in the concept of the continuum of care is the notion of functional linkages to care as well as between levels of care (e.g., from case identification to treatment and rehabilitation), including social protection systems [12]. When low-resourced settings are concerned, research tends to focus on linkage to care (e.g., detection, adherence), with less focus on the linkage of services across the care continuum.

## 3. An emphasis on Context

*“Women reported that it was considered inappropriate for them to walk on muddy roads and that they were afraid of slipping.”*

[13,14]

This single statement illustrates the great complexity in both the prevention and management of chronic diseases in what can be referred to as low-resource settings. In disentangling this single statement, one could reflect on multiple key factors that may influence health and health care, including gender or cultural bias (e.g., “inappropriate”), factors related to the natural and built environment (e.g., muddy roads), personal factors like self-efficacy (e.g., “afraid”), interpersonal factors such as social support, and health system challenges (e.g., inadequate emergency response services when injured due to slipping). Low-resource settings are typified by vast differences in the burden of disease and reflect underlying social determinants (e.g., income, education), causing widespread inconsistencies and systematic differences in prevalent disease clusters and health outcomes [15]. Importantly, low-resource settings are not endemic to LMIC (nor are high-resource settings to high-income countries), and vulnerable populations (e.g., migrants and ethnic minorities) in high-income countries may also face structural disparities and a disproportionate burden of disease. Global challenges, such as climate change, conflict, and migration may drive rapid demographic and contextual changes that can easily overburden existing healthcare resources and exacerbate inequities.

Further unravelling the concept of low-resource settings revealed nine thematic areas of resource constraints [15]. These included financial shortages, paucity of knowledge, underdeveloped infrastructure, restricted social resources, the influence of beliefs and practices, limitations in human resources, suboptimal healthcare delivery or governance, geographic or environmental challenges, and implications for research [15]. Many of these resource constraints influence health and health care at different levels of proximity to the patient (i.e., across the socio-ecological model) and across the continuum of care. For example, human resource constraints may imply challenges in social support (i.e., patient level) yet also workforce challenges specific to the management of prevalent health conditions (i.e., health system level). As such, resource constraints are generally not unidimensional or dichotomous (i.e., available versus not available) yet rather reflect a complex network of interrelated resource limitations spanning the socio-ecological model [15].

In addition to the nine themes areas proposed by van Zyl et al., other frameworks exist—mostly from the field of implementation science—that can aid the interrogation of context in relation to the study of complex health interventions. For example, the “Context and Implementation of Complex Interventions” (CICI) framework comprises three dimensions—context, implementation, and setting—which interact with one another and with the intervention dimension [16]. Within the CICI framework, context comprises seven domains (i.e., geographical, epidemiological, socio-cultural, socio-economic, ethical, legal, and political); implementation consists of five domains (i.e., implementation theory, process, strategies, agents, and outcomes); setting refers to the specific physical location, in which the intervention is put into practice. Particularly, the context and setting domain can be useful in structurally reflecting and reporting on pragmatic (implementation) research [16]. Alternatively, the widely used “Consolidated Framework for Implementation Research (CFIR) describes six domains, of which three domains (outer setting, inner setting, and individual) are of specific relevance when reflecting on context [17]. The CFIR is mostly used in the formative phase to identify determinants that may affect implementation outcomes yet also commonly contextualize research findings [18]. Using similar methods as van Zyl et al., Watson and colleagues describe eight constructs defining the external implementation context: (1) professional influences, (2) political support, (3) social climate, (4) local infrastructure, (5) policy and legal climate, (6) relational climate, (7) target population, and (8) funding and economic climate [19]. In addition, there are also frameworks that zoom into the factors relevant to understand inequity at a specific level, particularly personal characteristics. For example, the PROGRESS-Plus framework outlines personal characteristics that may contribute to inequity, including place, race/ethnicity, occupation, gender/sex, religion, education, socio-economic status, and social capital [20].

## 4. Call for Papers

As the effectiveness of complex interventions, as well as their success in reaching relevant populations, is critically influenced by their design and implementation in a given context, an in-depth and holistic understanding of the resource constraints (or availability) and their drivers within any setting is imperative [16]. However, in (global) health research, we often tend to sideline the significance of context or resort to umbrella terms to insinuate some wider applicability of our findings beyond the study’s context. The use of such terminology, without a structured interrogation, has been criticized, including terms like low-resource settings, low-to-middle-income countries, developing countries, or specific regions (e.g., sub-Saharan Africa) [21]. A call has been made to acknowledge and study the genuine uncertainty as to whether implementation strategies to deliver an evidence-based practice will be effective in a new context [22]. 

## 5. Summary

In summary, this Special Issue aims to collect a series of articles (primary or secondary research) that describe the current state or novel innovations that aim to understand or address pertinent chronic disease health challenges in low-resource settings (e.g., multimorbidity, non-communicable disease, communicable disease sequela, mental health, and disability). Ideally, the scope of the articles included in this Special Issue should equally reflect all stages of the continuum of care, ranging from primary prevention to rehabilitation and palliative care, including studies that focus on the functional linkages between levels of care in the health system. In parallel, we aim to challenge the practice of reporting study findings without a structured and in-depth reflection on the context in which the work (primary or secondary, quantitative or qualitative) took place [23]. Therefore, all submissions are strongly encouraged to include a separate yet consolidated exploration of context (max 300 words; see Box 1) based on the socio-ecological model as part of their submission. The socio-ecological model (as opposed to some other frameworks on theories) provides flexibility yet a structure independent of the type of research conducted. Upon completion, this Special Issue is expected to provide a unique collection of contextual evidence aimed to address pertinent health challenges in low-resource settings while providing shared learning on the context in which the research took place.

Box 1 **Exploration of contextual factors that may influence the conduct, outcome, or interpretation of the research findings based on Bronfenbrenner’ socio-ecological model (≤500 words) [24]. Ideally, factors that are included in the panel are supported by (original) data or existing literature.**

*Individual characteristics*

[Describe any individual characteristics (original data or literature) that may be specific or important to the context in which the research has been conducted]

*Micro-system*

[The microsystem represents the first layer in Bronfenbrenner’s socio-ecological theory and includes characteristics of the system that have direct contact with the individual in their immediate environment, such as family, religious organizations, health services, or community health workers]

*Meso-system*

[The mesosystem includes the interactions between different components within the individual’s microsystem. For example, if a caregiver of a patient interacts with a community health care worker, this may impact the health of the individual]

*Exo-system*

[The exo-system comprises formal and informal structures that do not “include” the individual but may indirectly affect the individual’s microsystem. For example, this may include features of health care, industry, social services, and local politics.]

*Macro-system*

[The macro-system historically comprises the cultural elements that implicitly affect the individual, including socio-economic status, wealth, poverty, and ethnicity. In addition, this layer comprises features of the built and natural environment that are implicit to the individual’s health outcomes, including neighborhood walkability, weather patterns, and the geography of the setting]

*Chrono-system*

[The chrono-system acknowledges changes that occur over a lifetime that may influ-ence development, including major life transitions (e.g., migration, parenthood) and historical events (e.g., apartheid, colonization).]

## Data Availability

Not applicable.
